# Effect of varying soil water potentials on methanogenesis in aerated marshland soils

**DOI:** 10.1038/s41598-017-14980-y

**Published:** 2017-10-31

**Authors:** Dirk Wagner

**Affiliations:** GFZ German Research Centre for Geosciences, Section 5.3 Geomicrobiology, Telegrafenberg, 14473 Potsdam, Germany

## Abstract

Wetlands are characterized by changing water tables, which have an influence on the activity of microorganisms. Particularly, the effect of oxygen on anaerobic methanogenic archaea is of importance for understanding greenhouse gas fluxes in wetlands. In this study the influence of oxygen on CH_4_ production in marshland soils was investigated in relation to varying soil water potentials. Water saturated samples as well as samples with drained macropores, and mesopores were used. Under anoxic conditions the CH_4_ production showed a dependence on the water content. The CH_4_ production rates varied between about 213 and 51 nmol g^−1^ soil h^−1^. In the presence of oxygen a correlation between CH_4_ production activity and water potential of the samples could not be demonstrated. Under oxic conditions with defined water potentials the CH_4_ production rates varied between about 141 and 58 nmol g^−1^ soil h^−1^. Cell counts of methanogenic archaea showed similar numbers in oxic and anoxic soil layers, and further illustrated living methanogens in the aerobic horizons of the marshland soil. The presented results are of great importance for modelling of the CH_4_ release from wetlands, because up to 25% of the CH_4_ is produced in the oxic horizon of the investigated marshland soil.

## Introduction

Methane (CH_4_) is one of the most potent greenhouse gases in the atmosphere and thus influencing global climate change. It contributes to the enhanced greenhouse effect with a portion of approximately 20% of all greenhouse gases^1^. The reason is the higher potential of methane for absorbing infrared radiation, which was currently new calculated with 28 to 34 times of that of CO_2_ over an integrated period of 100 years^2^. The concentration of atmospheric methane has drastically increased from 0.7 to 1.8 ppmv since pre-industrial times^3^. Seventy to eighty percent of this methane originates from microbial processes^4^. Thus, for the understanding of the recent and future CH_4_ dynamics in wetlands, it is important to deepen our knowledge of CH_4_ cycling microbial processes and the response of the microbial communities to changing environmental conditions.

Organic matter in soils and sediments are decomposed under anoxic conditions by a sequence of different groups of microorganisms within the anaerobic food chain. Methane formation (methanogenesis) is the last step of decomposition in hydromorphic environments. Besides enteric fermentation, natural wetlands (marshlands, fens, tundra, swamps) and rice paddies are the most important sources of atmospheric methane^5–7^. Although methane fluxes has been quantified for many terrestrial ecosystems^8–12^ little is known about the mechanisms controlling methane production in wetland soils^13–17^. Wetlands often show great spatial or temporal variations in environmental conditions which influences the release of CH_4_ into the atmosphere. Soil water content, temperature, type and amount of organic matter, vegetation, and the potential for methane oxidation are important factors^18–21^. For example groundwater table fluctuations are typical for wetlands such as marshes, and thus these soils are characterized by changing oxygen concentrations. However, methanogenic archaea are regarded as strictly anaerobic organisms. Growth and methane production in pure methanogenic cultures were observed only under anaerobic conditions^22^. Therefore, the influence of oxygen on the activity of methanogenic archaea is a key parameter for the understanding of methane fluxes in wetlands.

Little is known about the existence of methanogenic archaea in oxic environments such as forest floors, arable soils and aerobic layers of groundwater influenced wetlands. Despite being strictly anaerobic and non-sporeforming organisms, methanogenic archaea in pure cultures can survive from several hours up to 3 days following exposure to air^23,24^. Strains with high oxygen tolerance were isolated from ecosystems (e.g., permafrost soils) with changing oxygen conditions. However, few hours of contact with oxygen led to a rapid decrease in viability.

In contrast to pure cultures, the methanogenic community in natural soils survives well in microniches in otherwise oxygen influenced environments. Mayer and Conrad^25^ found a small methanogenic population in unsaturated paddy soils. Even in oxic desert soils, which are not commonly considered as habitats for anaerobic microorganisms, low cell numbers of methanogenic archaea were detected^26^. A rapid initiation of methane production in air-dried soil samples was reported, when they were incubated under submerged and anaerobic conditions^25^.

A previous study conducted with marshland soil from the same study site demonstrated a low methane production rate (0.35 to 2.43 nmol g^−1^ soil h^−1^) in the presence of oxygen^15^. Experiments with aerated slurries of these soils and different textured model soils, consisting of clay, sand and gravel, respectively, showed that the aerobic and facultative anaerobic microflora in association with soil particles such as clay formed anoxic microniches, and enable methane production in the presents of oxygen. The results suggested that a spatial coupling of aerobic respiration and anaerobic methane formation represent a well-established part of the methane production process in natural ecosystems influenced by changing oxygen concentrations.

In contrast to soil slurries, which were made of homogenized soil material, natural soils are characterized by a definite pore system. The microbial activity in soil environments is influenced by the soil water potential^27^. Together with the pore size distribution it decisively determines the part of gas-filled pore volume and consequently the concentration of oxygen affecting the activity of anaerobic methanogenic archaea. The aim of this study was to verify the effect of different water potentials on the methane production in marshland soils. For this purpose undisturbed soil samples from a marshland soil in Northern Germany were used analysing the methane production in dependence of the soil water potentials under oxic and anoxic conditions.

## Results

### Cell numbers of methanogenic archaea

**Figure 1:**
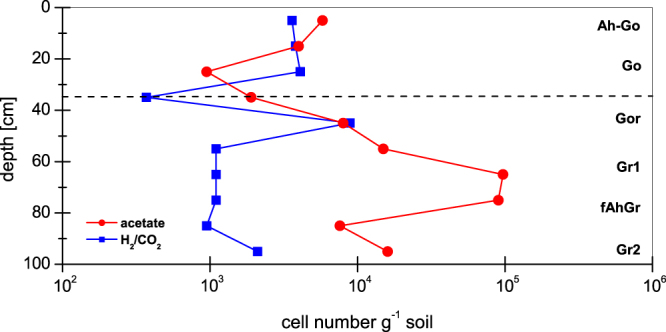
Vertical profile of the abundance of methanogenic archaea. MPN counts were determined in fresh marshland soil. The incubation was done at *in situ* temperature (10 °C) with acetate (20 mM) or H_2_/CO_2_ (80:20 v/v, pressurized 150 kPa) as substrate. The 95% confidence limits amounted to log 0.670 in a three-tube MPN analysis. The horizontal broken line indicates the groundwater table level.

Cell numbers of methanogenic archaea were determined at *in situ* temperature (10 °C). The vertical profile showed the distribution of methanogens within the investigated marshland soil for the main methanogenic substrates acetate and H_2_/CO_2_ (Fig. 1).

Methanogenic archaea were found in oxic (Go horizon) as well as in anoxic (Gr horizon) soil layers. Cell counts varied between 9.5 × 10^2^ to 9.7 × 10^4^ cells g^−1^ soil with acetate as substrate and between 3.7 × 10^2^ and 8.9 × 10^3^ cells g^−1^ soil with H_2_/CO_2_ as energy and carbon source. The average number of methanogens grown with H_2_/CO_2_ amounted to 2.7 × 10^3^ cells g^−1^ soil, whereas the cell counts with acetate were about 10 times higher (2.5 × 10^4^ cells g^−1^ soil).

Remarkable was that the cell numbers in oxic and anoxic soil layers were not significantly different, although methanogenic archaea are anaerobic organisms. The average number of methanogens in the upper 30 cm of soil amounted to 3.7 × 10^3^ cell g^−1^ soil and the counts of acetotrophic and hydrogenotrophic methanogenic archaea were similar. The average cell number from a soil depth of 30–100 cm was about 7 times higher (1.8 × 10^4^ cell g^−1^ soil) and acetotrophic methanogens occurred much more frequent.

### *In situ* CH_4_ production

**Figure 2:**
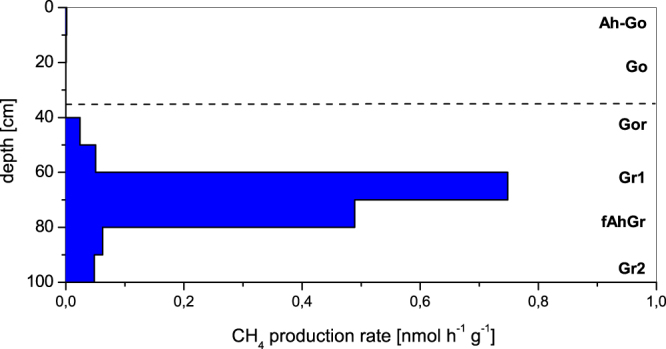
Vertical profile of *in situ* methane production activity. Methane production was analysed in fresh marshland soil. The incubation was done at *in situ* temperature (10 °C) without any additional substrate. The horizontal broken line indicates the groundwater table level.

Figure 2 shows the vertical profile of the CH_4_ production rates, which were analysed at *in situ* temperature (10 °C) without any additional substrate. CH_4_ production was determined for all soil layers between 40 and 100 cm depth. The rates in this zone fluctuate considerably between 0.02 and 0.75 nmol CH_4_ h^−1^ g^−1^ soil. The highest CH_4_ production rate was observed in the soil depth 60–70 cm (samples from this soil layer were therefore used for all other experiments). The results indicate that *in situ* methane production not only occurred in the reduced soil horizons (Gr, fAhGr) but also in the transition zone between oxic and anoxic conditions (Gor).

### Inhibition of CH_4_ oxidation by acetylene

For the study of methane production in the presence of oxygen soil material with the indigenous microflora was used. Besides methanogenic archaea this microflora included among other bacteria also methane-oxidizers, which convert methane to carbon dioxide. Therefore, it was necessary to inhibit the methane oxidation because otherwise it would lead to a falsification of the methane production activity in the experiment.

**Figure 3:**
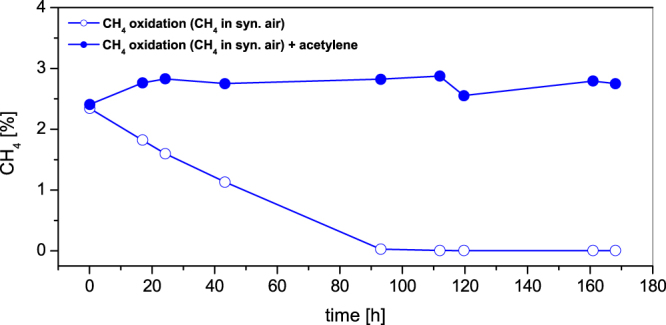
Influence of acetylene on methane oxidation in marshland soil samples. Soil slurries were incubated at 28 °C under a synthetic air atmosphere containing 2.5% CH_4_ in the presence and in the absence of acetylene.

The results showed that a methane concentration of 2.5% in synthetic air remained constant in the presence of acetylene. Without acetylene the methane in the headspace was completely oxidized within 95 h (Fig. 3). Additionally, the influence of acetylene on methane production under anoxic conditions with actetate as substrate was investigated. Without acetylene the methane production rate amounted to 46.5 nmol g^−1^ soil h^−1^ whereas the rate in the presence of acetylene was 30.3 nmol g^−1^ soil h^−1^ (results are not shown). This difference corresponded to the variation of methane production in natural soil samples.

These results indicated that the used acetylene concentration completely inhibited the methane oxidation without an influence on the methane formation.

### Methane production activity in relation to the soil water potential

**Figure 4:**
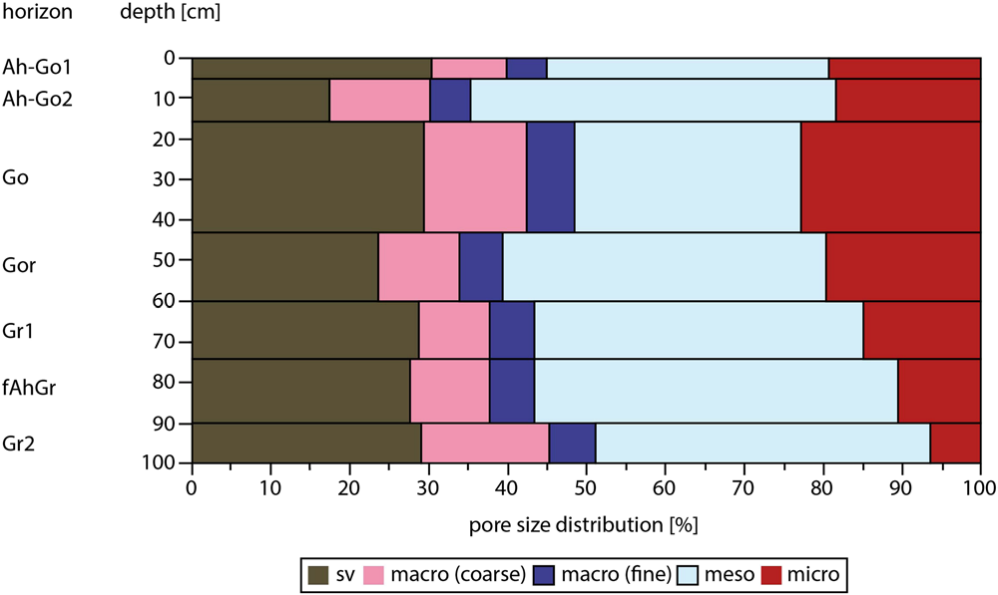
Vertical profile of pore size distribution of the marshland soil (sv = volume of soil matrix, macro = macropores, meso = mesopores, micro = micropores).

The soil matrix volume of the marsh amounted to an average of 26.6% and the total pore volume was 73.4%. The pore volume was differentiated in pore size ranges due to the following pore size distribution (Fig. 4): the coarse macropores (>50 µm) varied between 9.1 and 16.4% with an average of 11.7%, whereas the part of fine macropores (50–10 µm) was similar for each layer of the vertical profile (average of 5.4%). The total portion of macropores was 17.1%. The portion of mesopores (10–0.2 µm) amounted to an average of 40.2% and increased with the profile depth. The portion of micropores (<0.2 µm) varied in a large range between 6.5 and 22.9% (average 16.1%). Whereas the volume of the soil matrix and the distribution of maropores were homogeneous for the whole profile, the portion of micropores decreased in relation to the increase of the portion of mesopores.

**Figure 5:**
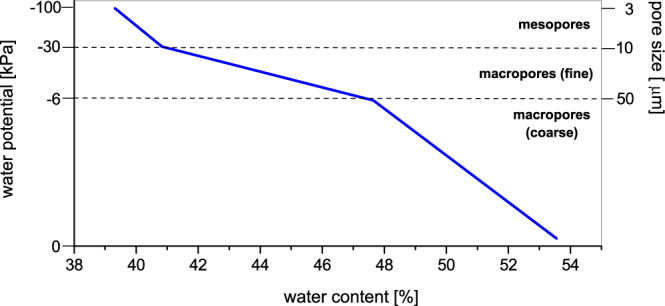
Soil water characteristic of undisturbed marshland soil samples for the drainage levels used in the experiments.

First of all, methane production of undisturbed soil samples was investigated under anoxic conditions to estimate the influence of the availability of water for methanogenic archaea. Therefore, different soil water potentials between 0 and −100 kPa were adjusted. On account of the required pressure it was possible to assign a definite part of the pore system to all drainage levels. The soil water characteristic (water retention curve) of undisturbed samples illustrated the connection between the soil water potential, the pore size, and the soil water content (Fig. 5). Marshland soil samples were water saturated at a water content of 54%. At a water potential of −6 kPa the samples had a water content of 48%, at −30 kPa the water content was 41% and at −100 kPa the water content decreased to 39%.

**Figure 6:**
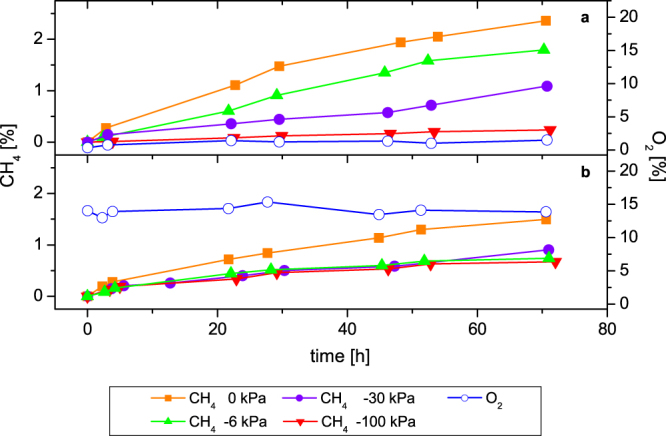
Progress of methane production of undisturbed soil samples related to different soil water potentials; (**a**) under anoxic and (**b**) under oxic conditions. As a substrate H_2_/CO_2_ (80:20 v/v) was used (n = 3 for anoxic conditions, n = 4 for oxic condition, for a better overview standard errors are not shown).

The progress of methane production of differently drained, undisturbed soil samples was almost linear under oxic and anoxic conditions for the whole incubation time (Fig. 6). The samples incubated with oxygen and acetylene showed a constant oxygen concentration of 15% O_2_ in the headspace.

**Figure 7:**
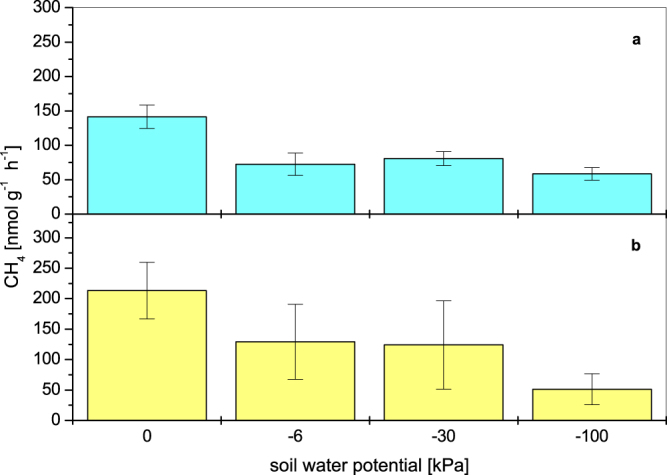
Methane production rates of undisturbed soil samples related to different soil water potentials; (**a**) under oxic and (**b**) under anoxic conditions. As a substrate H_2_/CO_2_ (80:20 v/v) was used (means ± standard error, anoxic conditions n = 3, oxic condition n = 4).

Despite of the high variation of methane production, the rates under anoxic conditions showed for each sample a dependence on the soil water potential (Fig. 7b, r = 0.957, *P* = 0.043). The highest methane production rate amounted to 213.37 ± 46.4 nmol g^−1^ soil h^−1^ in the samples with 100% water saturation and the lowest rate (51.27 ± 25.52 nmol g^−1^ soil h^−1^) was analysed for the high drained samples with a water potential of −100 kPa.

In the presence of oxygen the dependence of methane production activity in relation to the soil water potential, which was observed under anoxic conditions, was overlaid by the influence of oxygen on the anaerobic methanogenic archaea (Fig. 7a, r = 0.848, *P* = 0.152). The water saturated samples had a methane production rate of 141.28 ± 16.99 nmol g^−1^ soil h^−1^, whereas all other drainage levels showed only small differences in methane production between 58.43 ± 9.32 and 80.71 ± 10.18 nmol g^−1^ soil h^−1^.

A comparison of the methane production rates in the presence and in the absence of oxygen showed that the rates under oxic conditions for the water saturated soil samples reached about 66% of the rates under anoxic conditions (Fig. 7a,b). The methane production under oxic conditions of the other water potentials were about 33% compared to rates under anoxic conditions related to the water saturated soil samples. At a water potential of −6 and −30 kPa the methane production rates were only half in the presence of oxygen compared to the anoxic incubated samples. At a water potential of −100 kPa the methane production rate remained the same irrespective of oxygen levels. In the presence of oxygen no significant difference was found between methane production rates at water potentials between −6 and −100 kPa.

## Discussion

Some methanogenic archaea in soils can tolerate oxygen for a short period of time^23,24,28^, although these microorganisms are described as strictly anaerobic organisms. The presented results of methanogenic cell counts show, that these microorganisms were found within the whole profile of the marshland. Even in the upper oxic layer (Go horizon) about 10^3^ cells g^−1^ soil were determined with the MPN approach, which is also a validation for living and potential active methanogens. The detected cell numbers were only 7 times less than in the anoxic soil layers (Gr horizon). Methane production under *in situ* conditions at the time of sampling revealed activity in the oxic/anoxic transition zone and in the deeper soil layers with reduced redox conditions. However, a previous study demonstrated that even in aerated marshland and different textured model soils methane production in the presence of oxygen was possible^15^. A combination of biotic and abiotic soil parameters is responsible for the development of microniches and for protecting methanogenic archaea against oxygen.

Natural soils are characterized by a structure, which is assembled by the mineral and organic compounds and mostly by swelling and shrinking processes. The soil structure and the soil pore system are important for the water regime and the gas fluxes. The water content and the pore size distribution determines the water potential and the part of gas-filled pore volume and thus the influence of oxygen on the activity of anaerobic methanogenic archaea. Therefore, it was necessary to carry out the experiments with varying water potentials under aerobic and anaerobic conditions to investigate the influence of water potential independent from the influence of oxygen.

It is well known, that the activity of soil microorganisms is decisively controlled by the water potential^19^. The methane production activity under anoxic conditions of the undisturbed soil samples showed, according to the Kruskal-Wallis analysis, a significant dependence on the increasing water potential. In contrast, the methane production rates in the presence of oxygen for water potentials of −6, −30 and −100 kPa were not significant different. This indicates that the activity of methanogens in relation to the soil water potential under aerobic conditions was overlapped by the influence of oxygen. A step by step increase of the soil water potential should lead to a gradual decrease of the methane production activity.

The exchange of gases between soils and the atmosphere as well as the gas transport within soils differs due to the soil water potential because of different diffusion coefficients of water-filled and air-filled pores^29^. Oxygen for example is transported in the gas phase 10^4^ times faster (2.1 × 10^−1^ cm^−2^ s^−1^) than in the water phase (2.6 × 10^−5^ cm^−2^ s^−1^). The insignificant differences of methane production under oxic conditions indicated that the methanogenic archaea as well as the facultative anaerobic microorganisms had probably their greatest extent in the pores of 1 to 3 µm size, which were not drained in these experiments. The effect of oxygen was the same at all drainage levels and did not increase with increasing water potential. This interpretation is supported by the fact that the methane production rates of the high drained samples with a water potential of −100 kPa in the presence and in the absence of oxygen were similar.

This observation is in accordance with the results of Schricker^30^, who showed that the optimal water potential for microorganisms certainly was higher than −30 kPa, but a significant decline of the microbial activity was only observed when the pores ≤ 1 µm were drained. An influence of reduced substrate availability could be also excluded, because this effect can only be observed at a water potential of −600 kPa or higher^31^.

The results presented in a previous study by Wagner *et al*.^15^ and the data presented here, show, that methane production in aerobic horizons in wetland soils is possible by a spatial coupling with aerobic respiration. Only in combination with the absorbing capacity of soil particles such as clay, silt, and organic matter the indigenous microflora is able to develop a protective effect on the methanogenic archaea against oxygen. This potential of the investigated soil and the indigenous microbial community is of ecological significance because soils influenced by groundwater as well as other anaerobic habitats are periodically in contact with oxygen^32–35^. This particularly holds also for the investigated marshland soil, which is characterized by changing ground water tables.

**Figure 8:**
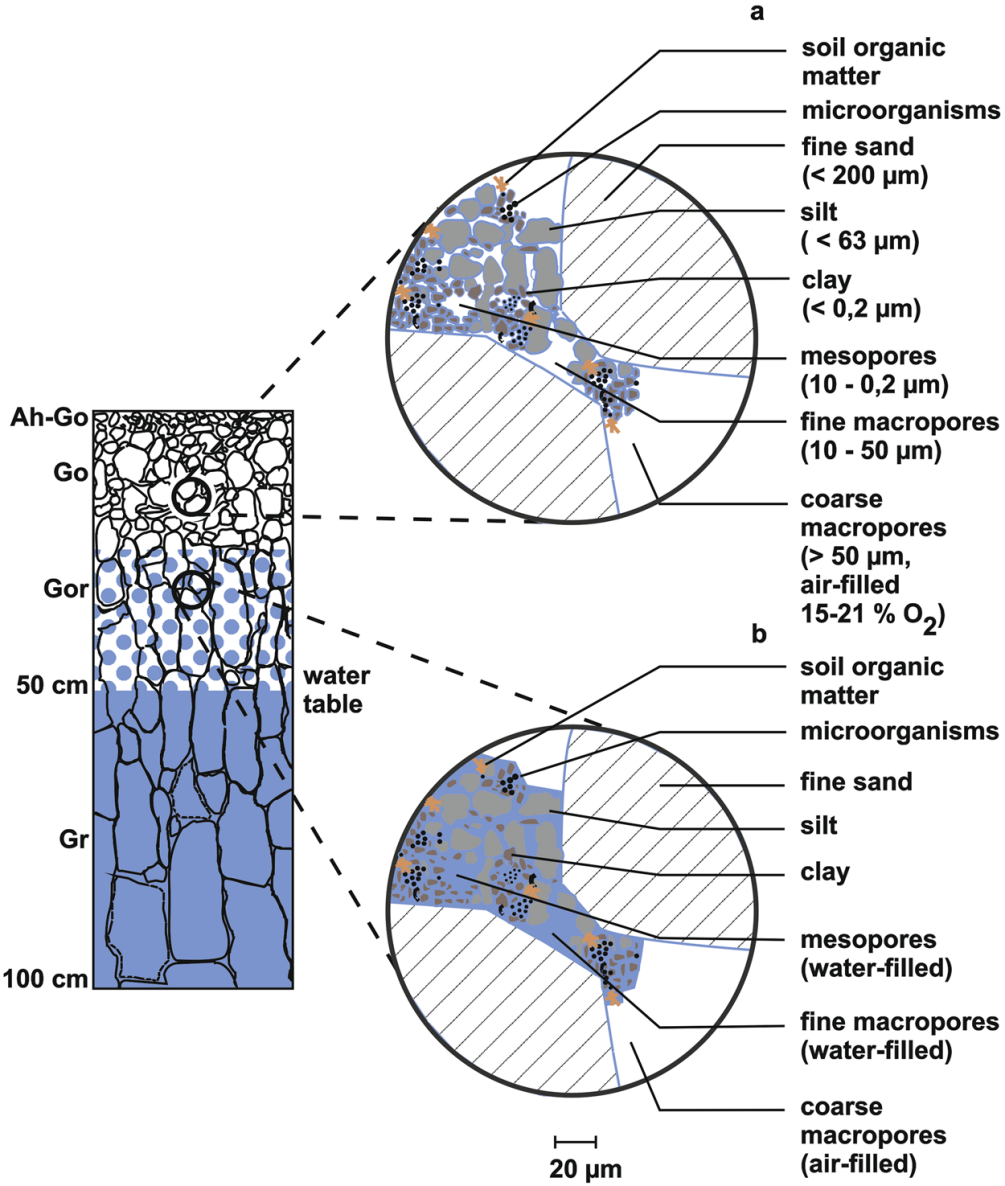
Descriptive model imaging the conditions for soil water and soil air in the investigated marshland soil at different water contents; (**a**) in the case when all pores (meso- and macropores) are drained (up to −100 kPa water potential), (**b**) in the case when only the coarse macropores are drained (−6 kPa water potential). The differences in the water content refer to the degree of filling of the pores (highlighted in blue).

Currently existing models for the calculation of methane release from wetlands often focusing on specific small-scale applications^36,37^ or oversimplifying the complexity of microbial processes, particularly the conditions under which methane production and oxidation may take place^38,39^. Best to our knowledge, there is only one process-based methane model published. It integrates for the first time the oxygen content into the modelling approach, in order to take into account methane oxidation potentials in periglacial landscapes^40^. However, of particular interest, is the potential activity of methanogenic archaea at the oxic-anoxic interface (Gor horizons) of marshlands shown in this study and eventually in other aerobic soils contributing to the methane production and therefore also to the methane emission. Figure 8 shows a descriptive model which integrates the soil water regime into the methane production potential in the presence of oxygen.

Below the water table (below 50 cm soil depth) all pores are filled with water and the diffusion of oxygen is mostly restricted (Eh~ -200 mV), which causes best conditions for methanogenic archaea and methane production. Above the water table the clay fraction, which had a portion of 20% in the investigated soil, stored enough water for biological processes within the Gor horizons^41^. At first, the macropores are drained under these conditions (Fig. 8b). At a pore oxygen concentration of 15–21% a sufficient supply of respiration processes are still guaranteed at a thickness of the water film of 1 cm^42^. The portion of coarse macropores of the investigated profile amounted to 10%. Therefore relatively high oxygen diffusion under these conditions is assumed. Despite this fact a more or less high methane production was determined, because the meso- and micropores, which are the preferred habitat of microorganisms, were water saturated. In these pores of the Gor horizon the diffusion of oxygen remains permanently restricted.

All coarse and fine macropores and the mesopores were drained in the Go horizons, especially during dry periods when the water table became very low. In this case only a small water film^43^ existed around sand and silt particles (Fig. 8a). An inhibition of the methanogenic archaea as a result of a higher oxygen transport rate and therefore a significant increase of methane oxidation were conceivable. Our results show, that under these circumstances (soil water/soil air conditions) 25% of the methane production rate in comparison of completely anoxic conditions will be obtained (Fig. 7). The presence of clay minerals enhances this potential. Under these drained conditions clay minerals are still swollen and reduce the oxygen diffusion to methanogens which are in close association with soil particles^41,44,45^.

## Conclusions

The investigations indicate, that methanogenic archaea survive not only in the aerobic layers of the marshland soil, but on account of their activity in an oxygen-influenced environment contribute also to methane production. Therefore, it is expected that other aerobic habitats such as forest floors and arable soils have a possible methane production potential and could be temporary a source of methane. The presented results are of great importance for modelling of methane release from natural wetlands, because this study shows that up to 25% of CH_4_ is produced in the oxic and transition layers (Go, Gor) of the investigated marshland soil, which can be a significant contribution to the methane production. This conclusion is supported by the studies of Walter^38^ for instance, who showed a difference of the calculated methane emission and the real determined methane release from North American wetlands, if the soils were not completely water saturated. Based on the presented results, it seems to be necessary to integrate the methane production activity in anaerobic niches in oxic soil horizons into future process-based methane modelling approaches.

## Methods

### Investigation site

The investigation area, which is called ‘Asseler Sand’ is located at the Lower Elbe near the city of Hamburg, Northern Germany (53°42′N, 9°27′E). The study site represents a typical freshwater marshland soil of perimarine silty loamy sediments. Further details on the properties of the investigated Elbe river marshland was given by Wagner *et al*.^15^.

### MPN counts

The number of methanogenic archaea was determined in a three-tube most probable number (MPN) analysis using a ten-fold serial dilution of soil in growth medium. The composition of the minimal medium was described by Wagner and Pfeiffer^46^.

The MPN-method was applied to count the cell numbers for a vertical profile of the investigated marshland. The vertical profile was determined to a depth of 100 cm. As energy and carbon source acetate (20 mM) or H_2_/CO_2_ (80:20 v/v, pressurized 150 kPa) were used. The bottles were incubated at 10 °C (*in situ* temperature at the time of sampling) for 12 weeks in darkness.

### Methane production activity

Fresh soil material (15 g) from 5 cm soil layers each was weighed into 25-ml glass jars and closed with black rubber stoppers. The samples were evacuated and flushed with ultra-pure N_2_. The prepared soil samples were incubated at 10°C without any additional substrate. Gas samples were taken every 24 h out of the jars headspace with a gastight syringe. CH_4_ production rates were calculated from the linear increase in CH_4_ concentration analysed by gas chromatography.

### Acetylene as a specific inhibitor for CH_4_ oxidation

To suppress CH_4_ oxidation in the presence of oxygen acetylene was used as a specific inhibitor^47^. A first test should show whether the CH_4_ oxidation activity was completely stopped with the used acetylene concentration and whether the CH_4_ production activity was influenced or not.

Therefore, fresh soil samples, taken from the anoxic layer (60–70 cm depth) of the profile, were passed through a stainless steel sieve of 2 mm mesh size. Then 100 g of the homogenized soil were weighed into 250 ml Erlenmeyer flasks and mixed with 20 ml sterile and anoxic tap water. These samples were used to study CH_4_ oxidation as well as CH_4_ production in the presence and in the absence of acetylene. In the case of CH_4_ oxidation the flasks were flushed with synthetic air containing 2.5% CH_4_, whereas the effect on CH_4_ production was investigated under anoxic conditions. In each case 3 samples were incubated with and without acetylene (60 nl acetylene ml^−1^ headspace). All slurries were shaken continuously at 28 °C. CH_4_ consumption and production was analysed during 170 h by gas chromatography.

### Pore size distribution and CH_4_ production at defined water potentials

The study of methane production activity in dependence on different soil water potentials was carried out using undisturbed soil samples taken with high-grade steel cylinder (100 cm^3^). The soils were sampled at a soil depth of 60–70 cm at the investigation site Asseler Sand. This soil horizon represented the layer with significant *in situ* methane production in the zone of changing groundwater table (Fig. 2).

To adjust water contents to specific pores (drained coarse macropores with < 50 µm, fine macropores of 50–10 µm and part of the mesopores with 10–3 µm) the undisturbed soil samples were drained using pressures of 0.3, 6, 30 and 100 kPa according to the method of Richards and Fireman^48^. To determine remaining pore size distribution disturbed soil material of above samples was also drained with a pressure of 300 and 1500 kPa. The total pore volume of the samples was analysed with a vacuum-air-pycnometer.

The 100 cm^3^ steel cylinder with the drained soil samples were placed into larger cylinders (Ø 10 cm, 35 cm hight, high grade steel) with a special fixture for 3 undisturbed soil samples^46^. The cylinders were closed with gastight seals at both ends. The top had two ports with ball taps. One port was used to exchange the headspace atmosphere and the other one had a screw cap with septum used as a syringe port.

The samples were used to study methane production of undisturbed marshland soils under oxic and anoxic conditions. In the case of anoxic methane production the cylinder was flushed with N_2_/CO_2_ (80:20 v/v). For the determination of methane production under oxic conditions the cylinder was flushed with synthetic air containing 20% oxygen. To inhibit methane oxidation, all samples (oxic and anoxic) were supplied with 60 nl acetylene ml^−1^ headspace. Additional H_2_/CO_2_ (20% H_2_, 100 kPa pressurized) was given as a substrate. Three replicates were used for the anoxic experiments and four replicates for the experiments under oxic conditions. All cylinders were incubated at 25 °C. Gas samples were taken from the headspace of the cylinder with a gastight syringe and analysed for the concentration of CH_4_ and O_2_ by gas chromatography. Methane production rates were calculated from the linear increase in methane concentration.

### Gas analysis

Methane and oxygen concentrations were determined with a Carlo Erba (GC 6000 vega series 2) gas chromatograph. The instrument was equipped with a Heyesep D (100/120 mesh, 20 ft) and a Molesieve 5 A (60/80 mesh, 7 ft) stainless steel column connected with a switching valve. Methane was analysed by a flame ionization detector (FID) and oxygen by a hot wire detector (HWD). All gas sample analyses were done after calibration with standards of known concentrations of the respective gases. The injector temperature was set at 100 °C, the columns at 70 °C and the FID at 200 °C. The detector temperature of the HWD was 100 °C and the filament cell temperature was 180 °C. Helium was used as carrier gas.

### Data Availability

The datasets generated during the current study are available from the corresponding author on reasonable request.
