# Author Correction: Effect of varying soil water potentials on methanogenesis in aerated marshland soils

**DOI:** 10.1038/s41598-018-25621-3

**Published:** 2018-05-09

**Authors:** Dirk Wagner

**Affiliations:** GFZ German Research Centre for Geosciences, Section 5.3 Geomicrobiology, Telegrafenberg, 14473 Potsdam, Germany

Correction to: *Scientific Reports* 10.1038/s41598-017-14980-y, published online 31 October 2017

The original version of this Article contained a typographical error in the Abstract.

“Under oxic conditions the CH4 production showed a dependence on the water content.”

now reads:

“Under anoxic conditions the CH4 production showed a dependence on the water content.”.

In addition, in the original version of this Article, Affiliation 1 was incorrectly listed as ‘GFZ German Research Centre for Geosciences, Section 5.3 Geomicrobiology, Telegrafenberg, 14469, Potsdam, Germany’. The correct affiliation is listed below:

GFZ German Research Centre for Geosciences, Section 5.3 Geomicrobiology, Telegrafenberg, 14473, Potsdam, Germany.

This has now been corrected in the PDF and HTML versions of the Article.

